# Protein and phosphoprotein levels in glioma and adenocarcinoma cell lines grown in normoxia and hypoxia in monolayer and three-dimensional cultures

**DOI:** 10.1186/1477-5956-10-5

**Published:** 2012-01-25

**Authors:** Victor A Levin, Sonali Panchabhai, Li Shen, Keith A Baggerly

**Affiliations:** 1 Department of Neuro-Oncology, The University of Texas MD Anderson Cancer Center, Houston, TX, USA; 2 Department of Bioinformatics and Computational Biology, The University of Texas MD Anderson Cancer Center, Houston, TX, USA

**Keywords:** glioblastoma, breast cancer, ovarian cancer, pancreatic cancer

## Abstract

**Background:**

Three dimensional (3D) growths of cancer cells in vitro are more reflective of in situ cancer cell growth than growth in monolayer (2D). The present study is designed to determine changes in protein and phosphoprotein that reflect adaptation of tumor cells to 3D as compared to 2D. Since relative hypoxia is a common feature of most solid tumors, the present study also aims to look at the impact of transition from normoxia to hypoxia in these two growth conditions.

**Results:**

Using reverse-phase protein arrays, we compared levels of 121 different phosphorylated and non-phosphorylated proteins in 5 glioma and 6 adenocarcinoma lines under conditions of 3D and monolayer culture in normoxia and hypoxia. A three-way analysis of variance showed levels of 82 antibodies differed between media (2D vs. 3D) and 49 differed between treatments (hypoxia vs. normoxia). Comparing 2D to 3D growth, 7 proteins were commonly (i.e., > 50% of tumors) elevated in 3D: FAK, AKT, Src, GSK3αβ, TSC2, p38, and NFκβp65. Conversely, 7 other proteins are commonly decreased: ATRIP, ATR, β-catenin, BCL-X, cyclin B1, Egr-1, and HIF-1α. Comparing normoxia to hypoxia, only NCKIPSD was commonly elevated in hypoxia; 6 proteins were decreased: cyclin B1, 4EBP1(Ser65), c-Myc, SMAD3(Ser423), S6(Ser235), and S6(Ser240). Hypoxia affected glioma cell lines differently from adenocarcinoma cell lines: 8 proteins were increased in gliomas (BAX, caspase 7, HIF-1α, c-JUN, MEK1, PARP 1 cleaved, Src, and VEGFR2) and none in adenocarcinomas.

**Conclusions:**

We identified subsets of proteins with clearly concordant/discordant behavior between gliomas and adenocarcinomas. In general, monolayer to 3D culture differences are clearer than normoxia to hypoxia differences, with anti-apoptotic, cytoskeletal rearrangement and cell survival pathways emphasized in the former and mTOR pathway, transcription, cell-cycle arrest modulation, and increased cell motility in the latter.

## Background

Cancer growth and invasion reflect many genetic and molecular events. These changes cannot be easily defined in situ, because (a) many factors are difficult to reproduce outside the host and (b) simplifications made to define variables with precision can create artifacts. In this and a prior study [1] we address a part of this problem. Specifically, we attempt to separate results due to a biological change of interest, the transition from normoxia to hypoxia, from those potentially induced by a simplification of the measurement process, growth in monolayer instead of in three dimensional cultures (3D). We have made other simplifications (e.g., using cell lines as opposed to primary cultures), so we are not perfectly "mimicking" disease conditions. Rather, we are focusing on effects of one specific simplification and outlining an approach that could be used more widely.

The importance of hypoxia to our understanding of tumor growth is based on the premise that all tumors, at some time, exhibit reduced oxygen delivery to the respiring neoplastic and stromal cells. This can be microscopic or macroscopic but can lead to proteome changes in neoplastic and stromal cells leading to impaired neoplastic growth through molecular mechanisms, resulting in cellular quiescence, differentiation, apoptosis, and necrosis [2,3] and activation of genes, transcription factors, proteins, and cytokine signals that can lead to regional tumor defensive strategies such as angiogenesis, anaerobic glycolysis, locomotion (invasion/metastasis), as well as tumor-specific survival strategies of apoptosis/autophagy [4,5]. These hypoxia-induced changes have presented challenges for cytotoxic chemotherapy and, likely, will do so for many targeted therapies. In addition, hypoxia diminishes the effectiveness of radiation therapy, in many cases, more for gliomas than for adenocarcinomas [6,7]. Thus, we hoped that being able to compare and contrast protein and phosphoprotein changes in glioma and adenocarcinoma cells might help design better treatment strategies for gliomas in the future.

The importance of studying protein changes in 3-dimensional (3D) growth is also important since a feature of malignant cells is their ability to grow in 3-dimensions (3D) as spheroids and colonies. This observation has led to greater study of tumors in 3D, as it is closer to in situ growth [8-11] even though it lacks many of the supporting extracellular systems (e.g., endothelial cells and capillaries, supporting matrices, cytokines, etc.). In addition, it has been observed that cancer cell lines grown in 2D and 3D culture respond differently to radiation and cytotoxic drugs [12-14]. Why do cell lines exhibit this differential behavior? Oxygenation of tumor cells also varies with 3D growth as cells grow distant from oxygen and nutrients, whether tumor cells are in 3D culture [15,16] or part of an in situ tumor [3,7,17,18]. Most studies of hypoxia in tumor cells have utilized 2D cultures [19,20].

In this study we begin to address the following questions. What protein and phosphoprotein changes reflect adaptations of tumor cells to 3D growth compared to 2D growth? What changes reflect adaptations from normoxia to hypoxia? Do tumor cells from high-grade glioma cell lines respond differently to 3D growth than adenocarcinoma cell lines? When exposed to relative hypoxic (aka microaerophilic) conditions, are changes in protein and phosphoprotein levels more affected by growth in 3D culture than they are by hypoxia?

In this study, we examine levels of 121 phosphorylated and non-phosphorylated proteins using reverse-phase protein array (RPPA) [1] technology. We examine these levels in eleven cell lines (including both gliomas and adenocarcinomas) under all combinations of media (2D and 3D) and growth conditions (normoxia and hypoxia), allowing us to properly relate changes to causes.

## Results and discussion

### Analysis using ANOVA (Three-Way Analysis of Variance)

**Figure 1:**
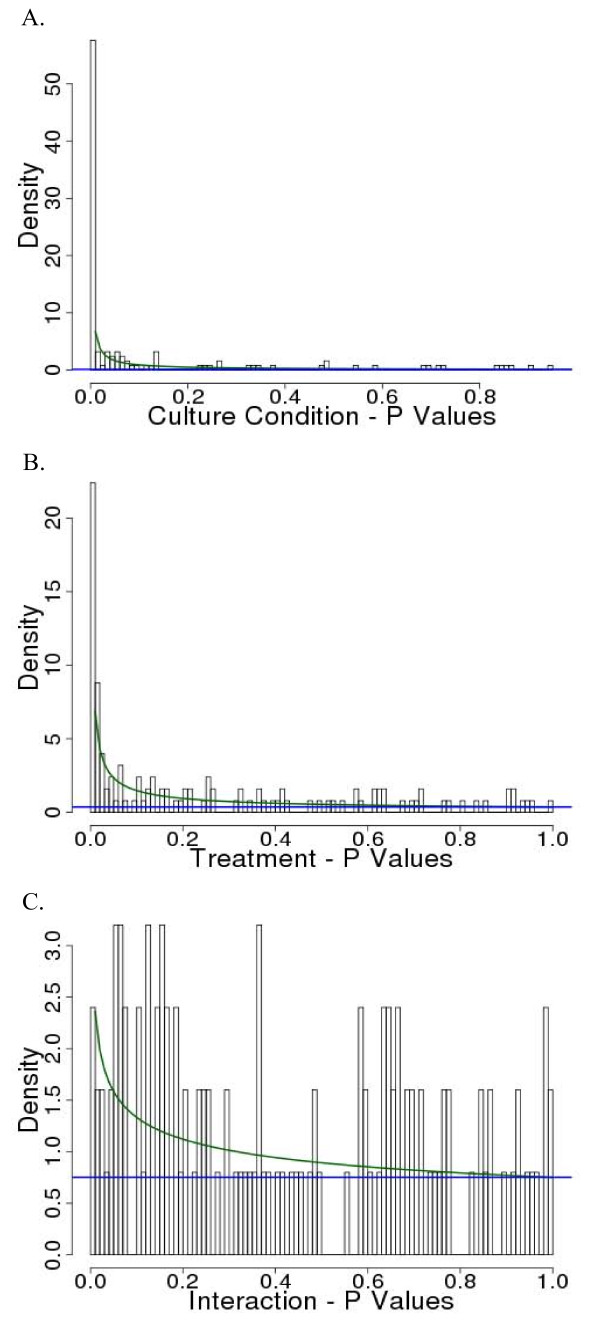
**Histogram showing the distribution of p-values from the feature-by-feature three-way ANOVAs**. Superimposed curves represent fits of the BUM models for (A) medium (2D and 3D), (B) treatment (hypoxia and normoxia), and (C) interaction between medium and treatment.

Our qualitative findings can be inferred from the p-value plots presented in Figure 1. Visual inspection of the distributions of p-values obtained for each ANOVA term (treatment, growth condition, cell line of origin, potential interaction between treatment and growth condition) clearly showed numbers of small p-values far greater than we would expect by chance for treatment, medium, and cell line, but not for the treatment-medium interaction (Figure 1). The cell line term is a nuisance factor, so we focused our attention on the individual effects of treatment and medium.

To account for multiple testing, we fit both distributions of p-values with beta-uniform mixture (BUM) models^5 ^and chose cutoffs to target false discovery rates (FDRs) of 5% and 1%. The extent of change (the height of the peak) is much more extensive for the shift from 2D to 3D than for the shift from normoxia to hypoxia. The corresponding plot for interaction terms here shows just a few significant alterations, suggesting that assessments of changes due to oxygenation conditions made in 2D are largely preserved in 3D, answering our primary question. However, the amount of change we see associated with the 2D-to-3D transition is so large that we feel quite uneasy about generalizing measurements from 2D in general without explicit testing. To determine what changes were "robust," we trichotomized residual terms for each effect (after correcting for others) by assigning scores of 1 (top 25%), -1 (bottom 25%), and 0 (all others), and summed these values by cell line or antibody, which is an approach we found useful in an earlier study [1]. We also used these sums to look for differences between gliomas and adenocarcinomas. No proteins showed a significant interaction between culture conditions and treatment in any cell line at the 5% FDR.

### Comparison of 2D and 3D Growth

**Figure 2:**
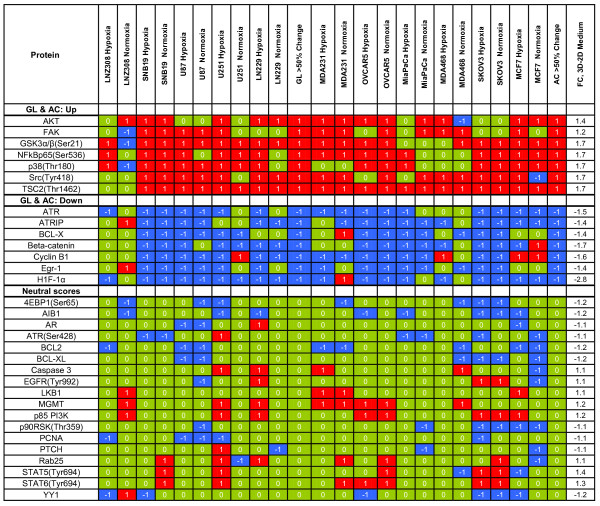
**Protein changes from ANOVA of 3D-2D (monolayer to 3D) medium by cell line whether normoxic or hypoxic conditions for proteins with p-value < 0.05**. Proteins are ordered from based on the FC (fold change) and scored -1, 0, or +1, depending on quartile distribution of raw antibody difference values (-1, blue, lowest quartile; +1, red, highest quartile; 0, green, others). Also shown is the score when > 50% of the total for glioma (GL; 6/10) and adenocarcinoma (AC; 7/12) values move in the same direction. The data is divided into six groupings based on > 50% score (+1, -1, and 0) for gliomas (GL) and adenocarcinomas (AC) together and separately as well as neutral and ill-defined scores.

**Figure 3:**
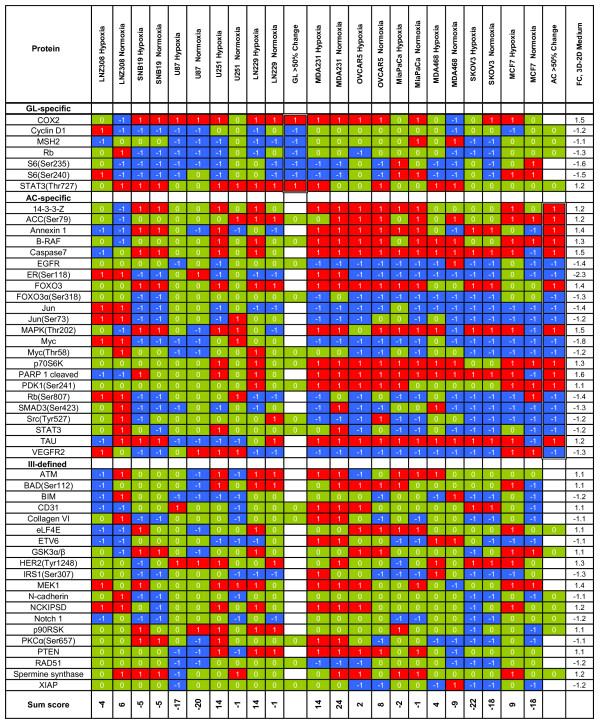
**Protein changes from ANOVA of 3D-2D (monolayer to 3D) medium by cell line whether normoxic or hypoxic conditions for proteins with p-value < 0.05**. Proteins are ordered from based on the FC (fold change) and scored -1, 0, or +1, depending on quartile distribution of raw antibody difference values (-1, blue, lowest quartile; +1, red, highest quartile; 0, green, others). Also shown is the score when > 50% of the total for glioma (GL; 6/10) and adenocarcinoma (AC; 7/12) values move in the same direction. The data is divided into six groupings based on > 50% score (+1, -1, and 0) for gliomas (GL) and adenocarcinomas (AC) together and separately as well as neutral and ill-defined scores. The Sum Score at the bottom of the figure is the summed values for columns in figures 2 and 3.

The comparisons that follow are the product of an aggregate analysis across 11 cell lines and 4 growth conditions focusing on the protein differences between 2D and 3D culture conditions. According to the BUM plots, 82 proteins were significantly different at a 5% FDR. Figures 2 and 3 show (a) the 3D-2D (change from 2D to 3D) sum scores with a focus on protein values from the ANOVA for proteins with p-values < 0.05, (b) the associated estimated fold changes in expression (negative values indicate expression was higher in 2D cultures), and (c) trichotomized scores for individual protein samples, broken down to show results for individual gliomas or adenocarcinomas (for all values see Additional file 1). Figures 2 and 3 entries are sorted by fold change, and overall sums of the robust scores by cell line are given at the bottom of the table. We also show the aggregate glioma and adenocarcinoma behavior by indicating whether the robust scores in a category showed consistent values for at least 50% of the samples examined. The glioma cell line most consistently changed by 3D-2D growth conditions was U87, with an average sum score across hypoxic/normoxic conditions of -18.5((-17+-20)/2) indicating protein and phosphoprotein down-regulation as conditions shift from 2D to 3D. By contrast, U251 ((14+-1)/2) and LN229 ((14+-1)/2) both showed general up-regulation of proteins when moving from 2D to 3D, though these gains were concentrated in the hypoxic conditions. For the adenocarcinomas, SKOV3 showed the greatest down regulation, with an average sum score of-20 ((-22+-18)/2), while MDA231 showed the greatest up regulation, with an average sum score of 19((14+24/2)).

Qualitative examination of Figures 2 and 3 shows that as a group, adenocarcinoma cell lines had 1.6 times more -1 sum scores and 2.0 times more +1 sum scores than glioma cell lines. However, approximately 32 proteins showed parallel changes in adenocarcinoma and glioma cell lines. The breakdown of these 32 proteins that moved in parallel in > 50% of glioma and > 50% of adenocarcinoma cell lines are as follows:

1) Levels of 7 proteins were lower in 3D than 2D cultures for the two groups: ATRIP, ATR, β-catenin, BCL-X, cyclin B1, Egr-1, and HIF-1α;

2) 18 proteins showed no grossly consistent differences: AIB1, AR, ATR(Ser428), BCL2, BCL-XL, caspase 3, EGFR(Tyr992), 4EBP1(Ser65), LKB1, MGMT, p85 PI3K, p90RSK(Thr359), PCNA, PTCH, Rab25, Stat6(Tyr694), Stat5(Tyr694) and YY1; and

3) Levels of 7 proteins were higher in 3D than 2D cultures: AKT, FAK, GSK3αβ(Ser21), NFκβp65(Ser536), p38(Thr180), c-Src(Tyr418), and TSC2(Thr1462).

In addition to the protein changes above, differences were seen between glioma and adenocarcinoma cell lines grown in 3D and 2D cultures. In glioma cell lines, protein or phosphoprotein levels of Stat3(Thr727) and COX2 were also higher in 3D cultures, whereas in adenocarcinoma lines, additional protein increases were seen in 14-3-3-Z, TAU, ACC(Ser79), annexin, caspase 7, FOXO3, MAPK(Thr202), p70S6K, B-RAF, PARP, and PDK1(Ser241). In glioma cell lines, lower protein level in 3D cultures was seen only for cyclin D1, MSH2, Rb, S6(Ser235), and S6(Ser240), whereas in adenocarcinoma lines, lower levels were seen in ER(Ser118), FOXO3α(Ser318), c-Jun(Ser73), c-Jun, c-Myc(Thr58), c-Myc, Rb(Ser807), SMAD3(Ser423), Src(Tyr527), Stat3, and VEGFR2.

### Comparison of Hypoxic and Normoxic Growth

**Figure 4:**
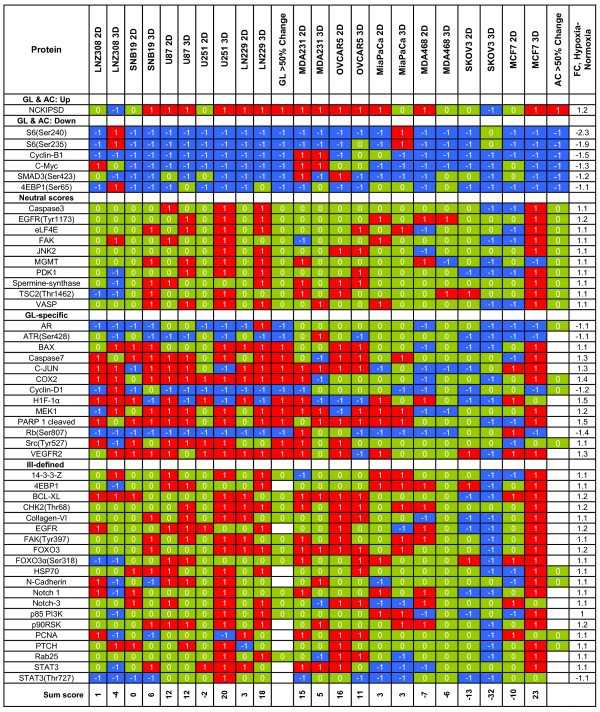
**Protein changes from ANOVA of the hypoxia-normoxia by cell line, whether grown in 2D or 3D conditions for proteins with p-values < 0.05**. The proteins are ordered based on the FC (fold change) and scored -1, 0, or +1 depending on quartile distribution of raw antibody difference values (-1, blue, lowest quartile; +1, red, highest quartile;0, green, others). Also shown is the score when > 50% of the total for glioma (GL; 6/10) and adenocarcinoma (AC; 7/12) values move in the same direction. The data is divided into five groupings based on > 50% score (+1, -1, and 0) for gliomas and adenocarcinomas together and separately as well as neutral and ill-defined scores.

The comparisons that follow are the product of an aggregate analysis across 11 cell lines and 4 growth conditions focusing on the protein differences between normoxia and hypoxia culture conditions. On the basis of the BUM plots, 50 proteins were significantly different in conditions of hypoxic and normoxic growth at a 5% FDR. Figure 4 focuses on (a) protein values from the ANOVA for proteins with p-values < 0.05, (b) the associated estimated fold change (negative values indicate that expression was higher in normoxic cultures), and (c) trichotomized scores for individual samples, broken down to show results for individual glioma and adenocarcinoma cell lines (for all values see Additional file 2). Figure 4 entries are sorted by fold change, and overall sums of the robust scores by cell line are given at the bottom. We have also shown aggregate glioma and adenocarcinoma behavior by indicating whether the robust scores in a category showed consistent values for at least 50% of the samples examined. Figure 4 shows that no glioma cell line showed a consistent decrease in sum scores between normoxic and hypoxic cultures, but some did increase. Protein and phosphoprotein sum scores were higher in hypoxic cultures for U87, LN229, and U251 cells, with sums of +12, +10.5, and +9, respectively. For the adenocarcinoma cell lines, SKOV3 had the most down regulation with an average sum score of -22.5 [(-13+-32)/2], while OVCAR5 and MDA231 showed the greatest up regulation, with average sum scores of 13.5 and 10, respectively.

Qualitative examination of Figure 4 shows that, as a group, glioma cells lines had 1.7 times more -1 sum scores and 10 times more +1 sum scores than adenocarcinoma cell lines. However, 17 proteins showed parallel changes in adenocarcinoma and glioma cell lines, as follows:

1) Levels of 6 proteins were lower in hypoxic conditions than in normoxic conditions in the two groups: cyclin B1, 4EBP1(Ser65), c-Myc, SMAD3(Ser423), S6(Ser235), and S6(Ser240);

2) 10 proteins showed no grossly consistent differences: caspase 3, EGFR(Tyr1173), elF4E, FAK, JNK2, MGMT, PDK1, spermine synthetase, TSC2(Thr1462), and VASP; and

3) 1 protein was higher in hypoxic cultures: NCKIPSD.

In addition to the protein changes reported above, differences were seen between glioma and adenocarcinoma cell lines grown in hypoxia and those grown in normoxia. In glioma cell lines, protein or phosphoprotein levels were also higher for BAX, caspase 7, HIF-1α, c-JUN, MEK1, cleaved PARP, Src(Tyr527), and VEGFR2, whereas no additional protein increases were seen in adenocarcinoma lines. In glioma cell lines, hypoxia caused declines in the expression of AR, ATR(Ser428), cyclin D1, and Rb(Ser807), whereas no additional protein decreases were seen in adenocarcinoma lines.

### Relevance of Protein Changes

In order to better understand the implications of the protein changes we observed, we used the Weizmann Institute of Science site http://www.genecards.org, Cell Signaling Technology http://www.cellsignal.com/, and TOCRIS Bioscience http://www.tocris.com/ to annotate the gene-associated proteins studied. Our interpretation of protein interactions and their implications is subject to a caveat: we only have an incomplete understanding of the non-linear interactions among signaling proteins, and, therefore, can only surmise functional significance from the protein changes we observed.

#### 2D to 3D Changes Overall

**Figure 5:**
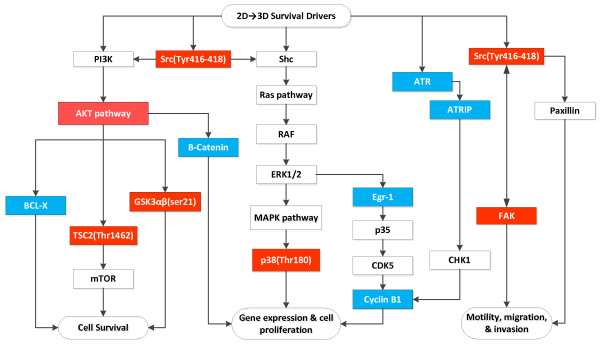
**This is a cartoon of the shared partial pathways involved in the transition of > 50% of glioma and adenocarcinoma cell lines from monolayer to 3D growth and are consistent with in situ tumors seeking to survive the more difficult 3D growth that will lead to genetic stress and part of the angiogenesis cascade**. The red color indicates the protein increased and the blue color that the protein decreased.

While there are a number of ways that our data could be analyzed and interpreted, we analyzed the aggregate data for 2D to 3D culture regardless of whether cells were grown in normoxia or hypoxia. From these data we concluded that the majority of cancer cell lines share some proteins that are increased to enable 3D growth and proteins that are reduced to minimize non-vital cell functions and focus. For the sake of discussion, and using available pathway analyses, we propose some relationships for the major protein changes observed for both glioma and adenocarcinoma cell lines (Figure 5). Increasing AKT can tend to decrease apoptosis and increase insulin stimulated protein synthesis by phosphorylating TSC2 (Thr1462) and activating mTOR signaling and phosphorylating 4E-BP1 and RPS6KB1. Increasing FAK, a substrate for c-Src, is important in cell migration and mobility, and appears to be important in shifting cancer cells from 2D to 3D growth. Similarly, increases in GSK3α/β should help in cell division and motility through its ability to phosphorylate signaling proteins, transcription factors, and structural proteins, all of which are needed to support 3D growth. Increases in NFκβ and p38, a MAP kinase family member, have effects on proliferation and transcriptional regulation through their ability to respond to cytokines and extracellular environmental stress, conditions that may be an advantage to cancer cells seeking to achieve 3D growth. Contra wise, the proteins levels that decreased suggested that these cancer cells did not need to protect themselves against DNA damage (ATR and ATRIP) or apoptosis (BCL-X and cyclin B1) or maintain cell adhesion on a plastic surface (β-catenin). The paradoxical decrease in transcriptional control of mitogenesis and differentiation (Egr-1) and HIF-1α is problematic. Even if we look at 2D to 3D growth separately for normoxia and hypoxia (see Additional File 1 for details) HIF1-α paradoxically decreased. Since HIF-1α did go up in the glioma lines in response to the shift from normoxia to hypoxia, it is possible that cells adapting to 3D growth in AlgiMatrix 3D Culture System or as a normal survival mechanism reduce HIF-1α protein or that HIF-1α degradation occurred under when cells were grown in the AlgiMatrix 3D Culture System in a manner similar to the ubiquitination seen with hypoxia-associated factor [21]. Thus, while we are confident of our finding, we are not sanguine as to its basis at this time since we did not measure the level of HIF-2α in our RPPA study. We have made our entire database available for others to mine (see Additional file 1 for details) in the expectation that scientists will find these data helpful and, possibly, better explain these findings.

#### 2D to 3D Changes Specific to Gliomas or Adenocarcinomas

In addition to the general changes, there were glioma-specific changes in protein levels. Increases in Stat 3 suggest that glioma cells, responding to cytokines and growth factors, activate transcription to help establish 3D growth. Increase in inducible COX2 is known to occur in gliomas and may, through prostanoid biosynthesis, enhance mitogenesis. There were also specific changes in adenocarcinoma lines. Increases may reflect increased signaling activities and direct effects on cell adhesion and anchorage-independent growth (14-3-3-Z), fatty acid synthesis (ACC), mediation of growth-regulated tyrosine kinases (annexin 1), regulation of MAPK/ERK signaling (B-RAF), activation of apoptosis (caspase 7, FOXO3), and transcription regulation and proliferation (MAPK). Interestingly, TAU, a microtubulin-associated protein, is differentially expressed in the nervous system and was thought to be somewhat unique to the nervous system, but we found higher levels of TAU in adenocarcinoma cell lines. What this means is not clear, but, given its effect in the nervous system, it may function to stabilize cytoskeletal proteins and be part of signaling system to organize adenocarcinoma cells in a basal-antral position for glandular functions.

#### Normoxia to Hypoxia Changes Overall

**Figure 6:**
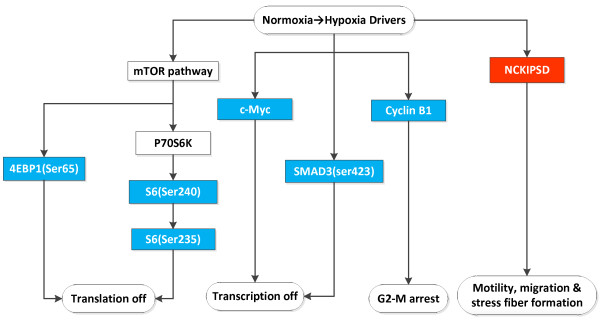
**This is a cartoon of the partial shared pathways involved in the transition of > 50% of glioma and adenocarcinoma cell lines from normoxia to hypoxia**. The red color indicates the protein increased and the blue color that the protein decreased.

Transitioning from normoxia to hypoxia, only 17 proteins move commonly among the glioma and adenocarcinoma cell lines. Interestingly, only one protein was elevated, NCKIPSD, a protein implicated in signal transduction as well as cell motility and stress fiber formation [22-27]. A relationship of the 6 proteins that decreased and the 1 protein that increased are depicted in Figure 6. In general, hypoxia appeared to decrease protein synthesis through the mTOR pathway to reduce cell cycle progression while supporting motility and migration through NCKIPSD

#### Normoxia to Hypoxia Changes Specific to Gliomas or Adenocarcinomas

**Figure 7:**
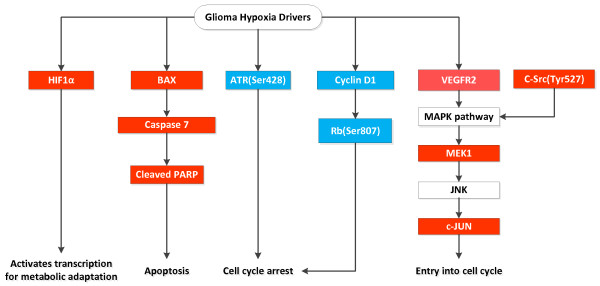
**This is a cartoon of the partial pathways involved in the transition of > 50% of glioma cell lines from normoxia to hypoxia**. The red color indicates the protein increased and the blue color that the protein decreased.

Glioma cell lines behave quite differently from adenocarcinoma cell lines when exposed to hypoxia. There are 8 increased proteins in gliomas (BAX, caspase 7, HIF-1α, c-JUN, MEK1, PARP 1 cleaved Src, and VEGFR2) and none in adenocarcinomas (Figure 7). It appears that gliomas are more responsive (sensitive) to hypoxia than adenocarcinoma. Both pro-survival and pro-apoptotic pathways are activated and a balance between these two might determine the ultimate outcome of the cells. The observations in glioma are consistent with the literature [28-31].

However, the fact that HIF-1α is not increased in adenocarcinoma cells is problematic with three possible explanations. 1) That hypoxic conditions were insufficient in the adenocarcinoma cell lines while sufficient in the gliomas to elicit activation of HIF-1α based on the possibility that astrocytes are constitutively more sensitive and responsive to hypoxia in keeping with their function to protect neurons and this functionality carries over to the glioma (malignant astrocytoma) tumors studied. 2) HIF-1α levels were constitutively up regulated (oncogenic) in the glioma lines independent of hypoxic effects. In an effort to answer this question, we compared each protein across cell lines and between glioma and adenocarcinoma groups from monolayer conditions. We found only 3 proteins that had a coefficient of variation of > 0.5 and T-test p < 0.05, and they were AKT(ser473), AK(Thr308) and HIF-1α; they were higher in base value in the glioma lines than adenocarcinoma lines by 4.7-, 3.0-, and 2.6-fold. Since the cell lines were compared to themselves, with respect to change in protein level, under the various "treatment" conditions of 3D culture and hypoxia, the differences in absolute level did not have an adverse effect on the data we report in Figures 1, 2, 3 and 4). 3) The failure of HIF-1α to increase with hypoxia in adenocarcinoma lines could be these cell lines mediate the hypoxia response mainly by HIF-2α orHIF-3α. Since we did not measure these proteins in our studies, we cannot test this hypothesis.

## Conclusion

We examined the proteins associated with each transition to see if there was clear involvement of specific pathways. Our findings were mixed. The changes are broad and extensive, not clearly concentrated in one area. During transition from 2D to 3D growth we see large changes at the protein level and AKT and MAPK pathways are mainly activated to provide survival and anticipated a need for angiogenesis in 3D. In hypoxia as compared to normoxia, the mTOR pathway is down-regulated. Also during hypoxia, when glioma cell lines are compared to adenocarcinomas, we infer that gliomas are much more responsive to hypoxia than adenocarcinomas as evident from the simultaneous apoptotic and pro-survival pathway activation.

We wish our experiments had definitively exposed a new therapeutic strategy for high-grade glioma and/or adenocarcinomas. What we have learned, however, is more tentative and incomplete. The mTOR pathways appeared to be down regulated in hypoxia in the current study and under conditions of starvation from our previous study. If mTOR pathways are normally down-regulated in tumor hypoxia, drug inhibitors of the mTOR pathway may not be a successful treatments for high-grade gliomas as the target may already be depressed, a conclusion supported by current clinical trials of mTOR inhibitors in glioblastoma patients [32-35].

Our data are available on the web. We hope others will look at our data and approach and make further observations to develop better chemotherapy strategies in the future.

## Methods

### Cell Lines

We used 11 established cell lines in this study. Six adenocarcinomas were comprised of three human breast cancer cell lines (MCF7, MDA231, MDA468), gifts from Francisco Esteva (MD Anderson); a human pancreatic carcinoma (MiaPaCa), a gift from Kapil Mehta (MD Anderson); and two human ovarian carcinomas (OVCAR5 and SKOV3), purchased from the American Type Culture Collection (Manassas, VA). Five high-grade glioma lines were comprised of U87, U251HF, and SNB19, bought from the American Type Culture Collection, and LNZ308 and LN229, gifts from Oliver Bogler (MD Anderson). Cells were maintained in Dulbecco's modified Eagle's medium: nutrient mixture F-12 (DMEM/F-12; Invitrogen, Carlsbad, CA) supplemented with10% fetal bovine serum (Sigma-Aldrich, St. Louis, MO) and 1% penicillin-streptomycin antibiotic (Invitrogen).

### Normoxia and Relative Hypoxia Conditions

For normoxia experiments, 2D and 3D cultured cells were incubated in a humidified incubator with constant supply of 5% CO2 at 37°C (21% oxygen). 2D and 3D cultures were grown under conditions of relative hypoxia using the Incubator Subchamber Culture System with the ProOx 110 oxygen controller (BioSpherix, Lacona, NY) that senses oxygen inside the chamber and maintains it at the set level that was, in our experiments, 1% oxygen for relative hypoxia (for 24 hours).

### Antibodies and Validation

The antibodies used are listed in Additional file 3, Table S1. To ensure that our antibodies were of sufficient quality, we used a denatured protein array and confirmed the specificity of the antibodies using Western blotting. Antibodies with only a single or dominant band on Western blotting were further assessed by direct comparison with RPPA using cell lines for differential protein expression, or they were modulated with ligands/inhibitors or siRNA for phosphoproteins or structural proteins, respectively. Only antibodies with Pearson correlations > 0.7 between RPPA and Western blotting were considered "validated" and used in this RPPA study. Antibodies were further assessed for specificity and quantification using phosphopeptides and non-phosphopeptides arrayed on nitrocellulose-coated slides; those with a second non-dominant band that could be rationalized and were otherwise consistent in terms of RPPA linearity they were used "with caution." As can be appreciated from the ***Numerical Preprocessing ***section below, the original study was initiated with 187 proteins to cover a large part of the known proteome, but because of technical issues, we were only able to study 121 different proteins in the RRPA.

### Preparation of Cell Lysates

The techniques used for the 2D studies were similar to those published previously [1,36], however, the isolation of cells from the 3D medium is described in detail here.

#### 2D Studies

Briefly, less than 10^6 ^cells/mL were plated in flasks, harvested in exponential growth at about 80% confluence, and harvested using 0.25% trypsin. Cells were counted with a Vi-Cell Counter (Beckman Coulter, Brea, CA), and 5 × 10^6 ^cells were transferred to six-well plates (35-mm diameter, 5-ml volume) that were grown for 24 h at 37°C in 5% CO_2 _and 20% O_2 _[1]. Cells were similarly cultured in parallel for 24 h in a 1% O_2 _hypoxic environment. Duplicate cultures were performed for each treatment. After 24 h, cells were washed in phosphate-buffered saline and lysed in 1% Triton X-100, 50 nM HEPES (pH 7.4), 150 mM NaCl, 1.5 mM MgCl_2_, 1 mM EGTA, 100 mM NaF, 10 mM sodium pyrophosphate, 1 mM Na_3_VO_4_, and 10% glycerol containing freshly added protease and phosphatase inhibitors. Cellular proteins were denatured by 1% sodium dodecyl sulfate (with ß-mercaptoethanol) and diluted in five serial 1:2 dilution steps using dilution buffer (lysis buffer containing 1% sodium dodecyl sulfate).

#### 3D Studies

We used the AlgiMatrix 3D Culture System six-well kit (Invitrogen), which is an animal origin-free bioscaffold that facilitates 3D cell culture. 2 × 10^4 ^cells in exponential growth were pipetted into six-well plates in 5 mL of medium (DMEM/F-12) and inoculated directly into the sterile microtiter plates preloaded with lyophilized alginate sponge that had been formulated using USP-grade raw material from brown seaweed, and each plate incubated at 37°C in an atmosphere of 5% CO_2 _and 20% O_2 _(i.e., normoxia) to allow the cells to form spheroids. The cell lines grew similarly except for the ovarian cancer cell lines that grew a bit slower.

On day 5, we transferred half the plates to the hypoxia chamber mentioned earlier and allowed them to grow for 24 h in relative hypoxia while the remaining half served as normoxia controls. To harvest spheroids after 24 h of hypoxia (i.e., on day 6), we followed the tri-sodium method described in the AlgiMatrix protocol. Briefly, 5 mL of pre-warmed iso-osmolar tri-sodium citrate solution was added to each well and incubated for 10 min at 37°C. The solution was prepared by diluting 55 mM tri-sodium citrate solution from 1 M stock solution, adding 1 g/L glucose, adjusting the osmolarity using 100 g/L NaCl solution, and adjusting the pH with 1 M citric acid solution to a pH of 7.2-7.4. After 10 min, the sponge biodegraded into the solution and the contents of each well was pipetted into a 15-mL centrifuge tube. To the tube, 5 mL of the same tri-sodium citrate solution was added, and the mixture was centrifuged for 7 min at 400 × g. The supernatant was removed, the pellet washed in phosphate-buffered saline to remove any remaining medium, and the pellet lysed using lysis buffer. The sample was then denatured, serially diluted, and arrayed on slides as in the 2D studies.

We manually isolated spheroids and determined the viability of single cells by adding them to 2 mL of trypsin-EDTA in a 15-mL tube, incubating at 37°C for a few minutes, agitating the tube for 15-20 min, and counting using the Vi-Cell cell viability analyzer (Beckman-Coulter). In all cases, the proportion of viable cells was greater than 90%.

### Array Assembly and Printing

Array assembly and printing were done as previously described. [37] In addition to the sample spots (2D normoxia, 2D hypoxia, 3D normoxia, and 3D hypoxia samples), each slide also included spots corresponding to positive and negative controls prepared from mixed cell lysates and dilution buffer, respectively. For quantification, protein lysates were passed through five serial 1:2 dilution steps, spotted in triplicate, and arrayed in 384-well plates (Genetix, Boston, MA). Samples were printed onto nitrocellulose-coated glass slides (FAST Slides, Schleicher & Schuell BioScience, Inc. USA, Keene, NH) using an Aushon BioSystems 2470 Arrayer (Aushon BioSystems, Inc., Burlington, MA) with 175-μm pins and a soft-touch deposition technology. For each triple, one series was located in the middle of the array and the other two were split on both sides and arranged in the reverse orientation, allowing us to estimate and correct for any spatial trends in intensity. To correct for staining, background, and loading variation across (array) slides, a positive control (a mixture of 12 cell lines hereafter called the "pooled control") and a lysate buffer negative control were printed at the end of each cell line sample row, creating a grid across the whole slide.

### Antibody Detection and Array Staining

Antibody and array staining were done as previously described [37,38]. Briefly, slides were probed with primary antibody plus a biotin-conjugated secondary antibody. The signal was amplified using a DakoCytomation-catalyzed system (Dako, Copenhagen, Denmark) and visualized by the diaminobenzidine colorimetric reaction. Slides were incubated for 15 min in biotin-blocking solution to block endogenous peroxidase, avidin, and biotin prior to incubating slides in protein block at 4°C overnight. Primary antibodies in concentrations from 1:100 to 1:2000 were added to the slides and allowed to remain for 1-2 h with frequent slide agitation to insure mixing on the slide (see supplement table S-1 for dilutions and manufacturers of antibodies). A biotinylated secondary antibody (anti-mouse or -rabbit), diluted 1:10000-1:20000 and used as a starting point for signal amplification, was added and allowed to remain in contact with the cells for 1 h. Subsequently, array slides were incubated using the Dako Signal Amplification System using a catalyzed reporter deposition of substrate to amplify the signal of the primary antibody. Slides were incubated in streptavidin-biotin-peroxidase and biotinyl tyramide/hydrogen peroxide reagents for 15 min each with washing in between the two incubations; 3,3'-diaminobenzidine tetrachloride was cleaved by tyramide-bound horseradish peroxidase, giving a stable brown precipitate.

### Analysis of RPPA Data

#### Experimental Design and Deviations

We studied 11 cell lines with two replicates under the four growth conditions resulting from combining 2D and 3D under normoxia and relative hypoxia, which would have ideally yielded 88 samples for measurement. Unfortunately, because of technical problems, there was only one replicate for LNZ308 in 3D under normoxia and hypoxia and one replicate for U87 in 3D in normoxia. Thus, we studied only 85 samples. Fortunately, the 41 pairs of exact replicates that did work are adequate to let us estimate the scale of technical variation, which is much smaller (variance 0.0053) than the variance 0.4615 for the cell line, growth condition, and treatment effects studied. Consequently, the replicate to replicate variation is sufficiently small and stable across our experiments relative to other sources of error that keeping the small number of samples without replicates will not lead to any distortion of the data.

#### Numerical Preprocessing

These samples were examined using 187 antibodies in RPPAs produced by the lead author's laboratory. Array images were produced using ImageQuant software (GE Healthcare, Waukesha, WI), and individual spot values were summarized using the MicroVigene RPPA module (VigeneTech, Carlisle, MA). After preprocessing was done, we used the R package SuperCurve (available at http://bioinformatics.mdanderson.org/Software/OOMPA) to summarize each five-step dilution series into one log_2 _scale (EC_50_) protein concentration value. The algorithm used fits a joint four-parameter logistic model [39]. Values for 153 of these arrays passed signal-to-noise filters assessed on control samples, giving the 85-by-153 data matrix we received from the core facility. Rows of this matrix (samples) were centered on the median to adjust for potential differences in sample loading. Correlations between replicate spotting's of the same samples on each array were also checked for consistency; we retained only the 124 (121 different proteins with 3 duplicates listed by different names) that showed correlations in excess of 0.5. Clustering and other exploratory data analysis showed that labels for samples 63 (MDA468-3D Hypoxia) and 83 (MDA231-3D Hypoxia) had accidentally been swapped; we corrected this.

#### Statistical Analysis

We used three-way ANOVAs, protein by protein, to model the log_2 _expression values produced by the RPPAs. We included terms for treatment (hypoxia or normoxia), growth conditions (2D or 3D), and cell line of origin. We also included a term to account for potential interaction between treatment and medium.

To account for multiple testing, we fit distributions of p-values for each contrast with beta-uniform mixture (BUM) models^5 ^and chose cutoffs to target false discovery rates (FDRs) of 5% and 1%.

To determine what changes were "robust," we trichotomized residual terms for each effect (after correcting for others) by assigning scores of 1 (top 25%), -1 (bottom 25%), and 0 (all others), and summed these values by cell line or antibody, which is an approach we found useful in an earlier study [1]. We also used these sums to look for differences between gliomas and adenocarcinomas. Full details of our analyses (including data and code) are available from http://bioinformatics.mdanderson.org/Supplements/LevinHypoxia/ 

## Competing interests

The authors declare that they have no competing interests.

## Authors' contributions

VAL conceived of the project, obtained funding for the research, and was the primary author. SP was the post-doctoral student who conducted the main aspects of the study and helped write the paper. KAB and LS did the RPPA analyses and the statistical portions of this study. KAB also helped write the paper. All authors approved the final manuscript.

## Supplementary Material

Additional file 1 **This file contains is from the quantile matrix of trichotomized values for 3D-2D values used in Figures **2** and **3** of the paper**. Click here for file

Additional file 2 **This file contains is from the quantile matrix of trichotomized values for hypoxia-normoxia values used in Figure **4** of the paper**. Click here for file

Additional file 3 **Table S1**. Descriptions of 121 Distinct and 3 Duplicate Antibodies Used in Our RPPA Studies. BCL-XL, collagen VI, and Src(Tyr416/418), were used in duplicate, bringing the total number of antibodies studied in the RPPA to 124. Phosphorylation sites are indicated in parentheses. Click here for file
