# Supplementary material for: Protein and phosphoprotein levels in glioma and adenocarcinoma cell lines grown in normoxia and hypoxia in monolayer and three-dimensional cultures
Source: Proteome Sci. 2012 Jan 25;10:5. doi: 10.1186/1477-5956-10-5 (PMC3317865; doi:10.1186/1477-5956-10-5)
Supplement: Additional file 3 — Table S1. Descriptions of 121 Distinct and 3 Duplicate Antibodies Used in Our RPPA Studies. BCL-XL, collagen VI, and Src(Tyr416/418), were used in duplicate, bringing the total number of antibodies studied in the RPPA to 124. Phosphorylation sites are indicated in parentheses. [file 1477-5956-10-5-S3.DOCX]

**Table S1**. Descriptions of 121 Distinct and 3 Duplicate Antibodies Used in Our RPPA Studies. BCL-XL, collagen VI, and Src(Tyr416/418), were used in duplicate, bringing the total number of antibodies studied in the RPPA to 124. Phosphorylation sites are indicated in parentheses.

| **Name** | **Catalogue Number** | **Source** | **Species** |
| --- | --- | --- | --- |
| 14-3-3-Z | 1019 | Santa Cruz | Rabbit |
| 4EBP1 | 9452 | Cell Signaling | Rabbit |
| 4EBP1(Ser65) | 9456 | Cell Signaling | Rabbit |
| 4EBP1(Thr37) | 9459 | Cell Signaling | Rabbit |
| 4EBP1(Thr70) | 9455 | Cell Signaling | Rabbit |
| ACC(Ser79) | 3661 | Cell Signaling | Rabbit |
| AIB1 | 611105 | BD Biosciences | Mouse |
| AKT | 9272 | Cell Signaling | Rabbit |
| AKT(Ser473) | 9271 | Cell Signaling | Rabbit |
| AKT(Thr308) | 9275 | Cell Signaling | Rabbit |
| AMPKα | 2532 | Cell Signaling | Rabbit |
| Annexin 1 | 71-3400 | Invitrogen | Rabbit |
| AR | 1852 | Epitomics | Rabbit |
| ATM | 35420 | AbCam | Rabbit |
| ATR | 2790 | Cell Signaling | Rabbit |
| ATR(Ser428) | 2853 | Cell Signaling | Rabbit |
| ATRIP | 2737 | Cell Signaling | Rabbit |
| BAD(Ser112) | 9291 | Cell Signaling | Rabbit |
| BAX | 2772 | Cell Signaling | Rabbit |
| BCL2 | M0887 | Dako Cytomation | Mouse |
| BCL-X | 1018-1 | Epitomics | Rabbit |
| BCL-XL | 2762 | Cell Signaling | Rabbit |
| Beta-catenin | 9562 | Cell Signaling | Rabbit |
| BIM | 1036 | Epitomics | Rabbit |
| B-RAF | 1647 | Epitomics | Mouse |
| Caspase 3 | 1476-1 | Epitomics | Rabbit |
| Caspase 7 | -9491 | Cell Signaling | Rabbit |
| CD31 | M0823 | Dako Cytomation | Mouse |
| CHK2 | 3440 | Cell Signaling | Mouse |
| CHK2(Thr68) | 2197 | Cell Signaling | Rabbit |
| c-Jun | 9165 | Cell Signaling | Rabbit |
| c-Jun(Ser73) | 9164 | Cell Signaling | Rabbit |
| c-Kit | 1522 | Epitomics | Rabbit |
| c-Myc | 9402 | Cell Signaling | Rabbit |
| c-Myc(Thr58) | 9401 | Cell Signaling | Rabbit |
| Collagen VI | 20649 | Santa Cruz | Rabbit |
| COX2 | 2169-1 | Epitomics | Rabbit |
| Cyclin B1 | 1495 | Epitomics | Rabbit |
| Cyclin D1 | 718 | Santa Cruz | Rabbit |
| Cyclin E1 | 247 | Santa Cruz | Mouse |
| E-cadherin | 4065 | Cell Signaling | Rabbit |
| EGFR | 3 | Santa Cruz | Rabbit |
| EGFR(Tyr1173) | 1124 | Epitomics | Rabbit |
| EGFR(Tyr992) | 2235 | Cell Signaling | Rabbit |
| Egr-1 p82 | 110 | Santa Cruz | Rabbit |
| eLF4E | 9742 | Cell Signaling | Rabbit |
| ERCC1 | MS-671-PO | Lab Vision | Rabbit |
| ERα(Ser118) | 1091 | Epitomics | Rabbit |
| ETV6 | GA2101 | Strategic Diagnostics |  |
| FAK | 1700 | Epitomics | Rabbit |
| FAK(Tyr397) | 3283 | Cell Signaling | Rabbit |
| FOXO3 | 9467 | Cell Signaling | Rabbit |
| FOXO3α(Ser318) | 9465 | Cell Signaling | Rabbit |
| GATA3 | 558686 | BD Biosciences | Mouse |
| GSK3α/β | 7291 | Santa Cruz | Mouse |
| GSK3α/β(Ser21) | 9331 | Cell Signaling | Rabbit |
| HER2 | 2242 | Cell Signaling | Rabbit |
| HER2(Tyr1248) | 06-229 | Upstate (Millipore) | Rabbit |
| HIF-1α | 80544 | AbCam | Mouse |
| hSAMDC | Custom made | David Feith lab | Rabbit |
| HSP27 | 2402 | Cell Signaling | Mouse |
| HSP70 | 4872 | Cell Signaling | Rabbit |
| IGFBP2 | 3922 | Cell Signaling | Rabbit |
| IRS1 | 06-248 | United Biochemical | Rabbit |
| IRS-1(Ser307) | 1194 | AbCam | Rabbit |
| JNK2 | 4672 | Cell Signaling | Mouse |
| KU80 | 2180 | Cell Signaling | Rabbit |
| LKB1 | 15095 | AbCam | Mouse |
| MAPK(Thr202) | 4377 | Cell Signaling | Rabbit |
| MEK1 | 1235 | Epitomics | Rabbit |
| MEK1/2(Ser217) | 9121 | Cell Signaling | Rabbit |
| MGMT | 16200 | Chemicon | Mouse |
| MSH2 | 2850 | Cell Signaling | Mouse |
| N-cadherin | 4061 | Cell Signaling | Rabbit |
| NCKIPSD | 2117 | Strategic Diagnostics | Rabbit |
| NFκBp65(Ser536) | 3033 | Cell Signaling | Rabbit |
| Notch 1 | 32745 | Santa Cruz | Mouse |
| Notch 3 | 5593 | Santa Cruz | Rabbit |
| p27 | 1591 | Epitomics | Rabbit |
| p38(Thr180) | 9211 | Cell Signaling | Rabbit |
| p53 | 9282 | Cell Signaling | Rabbit |
| p70S6K | 1494 | Epitomics | Rabbit |
| p90RSK | 9347 | Cell Signaling | Rabbit |
| p90RSK(Thr359) | 9344 | Cell Signaling | Mouse |
| PARP 1 cleaved | 9546 | Cell Signaling | Rabbit |
| PAX2 | 1500-1 | Epitomics | Rabbit |
| PCNA | 29 | AbCam | Mouse |
| PDK1 | 3062 | Cell Signaling | Rabbit |
| PDK1(Ser241) | 3061 | Cell Signaling | Rabbit |
| PI3K p85 | 06-195 | Upstate (Millipore) | Rabbit |
| PI3K-p110a | 4255 | Cell Signaling | Rabbit |
| PKCa(Ser657) | 06-822 | Upstate (Millipore) | Rabbit |
| PKCα | 05-154 | Upstate (Millipore) | Mouse |
| PR | 1483 | Epitomics | Rabbit |
| PTCH | 2113 | Strategic Diagnostics | Rabbit |
| PTEN | 9552 | Cell Signaling | Rabbit |
| Rab25 | Custom made | Covance | Mouse |
| RAD51 | na 71 | Chem Biotech | Mouse |
| Rb | 9309 | Cell Signaling | Mouse |
| Rb(Ser807) | 9308 | Cell Signaling | Rabbit |
| S6 | 2217 | Cell Signaling | Rabbit |
| S6(Ser235) | 2211 | Cell Signaling | Rabbit |
| S6(Ser240) | 2215 | Cell Signaling | Rabbit |
| SMAD3 | 1735 | Epitomics | Rabbit |
| SMAD3(Ser423) | 9520 | Cell Signaling | Rabbit |
| Spermine-synthase | Custom made | David Feith lab | Rabbit |
| c-Src(Tyr416) | 2101 | Cell Signaling | Rabbit |
| c-Src(Tyr527) | 2105 | Cell Signaling | Rabbit |
| STAT3 | 06-596 | Upstate (Millipore) | Rabbit |
| STAT3(Thr727) | 9134 | Cell Signaling | Rabbit |
| STAT3(Tyr705) | 9131 | Cell Signaling | Rabbit |
| STAT5 | 1289 | Epitomics | Rabbit |
| STAT5(Tyr694) | 9314 | Cell Signaling | Rabbit |
| STAT6(Tyr641) | 9361 | Cell Signaling | Rabbit |
| TAU | 05-348 | Upstate (Millipore) | Mouse |
| TAZ | 3961 | AbCam | Rabbit |
| Tuberin/TSC2(Thr1462) | 1613-1 | Epitomics | Rabbit |
| VASP | 3112 | Cell Signaling | Rabbit |
| VEGFR2 | 2479 | Cell Signaling | Rabbit |
| XIAP | 2042 | Cell Signaling | Rabbit |
| YY1 | 2185 | Cell Signaling | Rabbit |
